# Anti-Inflammatory and Neuroprotective Constituents from the Peels of *Citrus grandis*

**DOI:** 10.3390/molecules22060967

**Published:** 2017-06-09

**Authors:** Ping-Chung Kuo, Yu-Ren Liao, Hsin-Yi Hung, Chia-Wei Chuang, Tsong-Long Hwang, Shiow-Chyn Huang, Young-Ji Shiao, Daih-Huang Kuo, Tian-Shung Wu

**Affiliations:** 1 School of Pharmacy, College of Medicine, National Cheng Kung University, Tainan 701, Taiwan; z10502016@email.ncku.edu.tw (P.-C.K.); truthloveroy@yahoo.com.tw (Y.-R.L.); z10308005@email.ncku.edu.tw (H.-Y.H.); 2 Department of Chemistry, National Cheng Kung University, Tainan 701, Taiwan; w96s062001@hotmail.com; 3 Graduate Institute of Natural Products, College of Medicine, Chang Gung University, Taoyuan 333, Taiwan; htl@mail.cgu.edu.tw; 4 Research Center for Chinese Herbal Medicine, Research Center for Food and Cosmetic Safety, and Graduate Institute of Health Industry Technology, College of Human Ecology, Chang Gung University of Science and Technology, Taoyuan 333, Taiwan; 5 Department of Anesthesiology, Chang Gung Memorial Hospital, Taoyuan 333, Taiwan; 6 Department of Pharmacy, Chia-Nan University of Pharmacy and Science, Tainan 717, Taiwan; schuang@mail.cnu.edu.tw; 7 Division of Basic Chinese Medicine, National Research Institute of Chinese Medicine, Ministry of Health and Welfare, Taipei 112, Taiwan; yshiao@nricm.edu.tw; 8 Department of Pharmacy, College of Pharmacy and Health Care, Tajen University, Pingtung 907, Taiwan; dhkou@tajen.edu.tw

**Keywords:** *Citrus grandis*, chromatographic method, spectroscopic elucidation, superoxide anion generation, elastase release, neuroprotective

## Abstract

A series of chromatographic separations performed on the ethanol extracts of the peels of *Citrus grandis* has led to the characterization of forty compounds, including seventeen coumarins, eight flavonoids, two triterpenoids, four benzenoids, two steroids, one lignan, one amide, and five other compounds, respectively. The chemical structures of the purified constituents were identified on the basis of spectroscopic elucidation, including 1D- and 2D-NMR, UV, IR, and mass spectrometric analysis. Most of the isolated compounds were examined for their inhibition of superoxide anion generation and elastase release by human neutrophils. Among the isolates, isomeranzin (**3**), 17,18-dihydroxybergamottin (**12**), epoxybergamottin (**13**), rhoifolin (**19**), vitexicarpin (**22**) and 4-hydroxybenzaldehyde (**29**) displayed the most significant inhibition of superoxide anion generation and elastase release with IC_50_ values ranged from 0.54 to 7.57 μM, and 0.43 to 4.33 μM, respectively. In addition, 7-hydroxy-8-(2′-hydroxy-3′-methylbut-3′-enyl)coumarin (**8**) and 17,18-dihydroxybergamottin (**12**) also exhibited the protection of neurons against Aβ-mediated neurotoxicity at 50 μM.

## 1. Introduction

The genus *Citrus* is composed of various species grown all over the tropical and subtropical regions. Each species has its own characteristic aroma and some *Citrus* peel oils have been used in a variety of products such as foods, beverages and perfumes [1]. A lot of scientific reports have been published regarding the compositions and significant bioactivity of the *Citrus* peels. *C. grandis* belongs to the family Rutaceae, which is cultivated throughout Taiwan, Indochina, and Southern China [2,3,4,5,6,7,8,9,10,11]. The peels of mature fruits of *C. grandis* have been used in traditional Chinese medicine for treating the common cold and cancer, and relieving exhaustion [12]. Many natural products such as flavonoids, coumarins, and terpenoids have been isolated from *C. grandis* [2,3,4,5,6,7,8,9,10,11]. Among these, various principles have been reported to exhibit antioxidant [13,14,15,16], anti-inflammatory [16,17,18,19,20], anti-microbial [21,22,23,24], and anti-cancer effects [25,26,27,28]. In addition, several compounds were subjected to the study related to the prevention of vascular diseases [29,30,31,32]. The constituents of the peels of *C. grandis* were found to be rich in coumarins and flavonoids. The substitution of multi-methoxyl group of flavonoids, polymethoxyflavones (PMFs) were found to be rich in *Citrus* which have been reported to exhibit antioxidant because of the delocalized electrons [33]. That is why the *Citrus* genus is an important food source for people, and it was also an important Chinese medicine from ancient times. In a previous study [34], *Citrus* flavonoids were reported to exhibit anti-inflammatory and neuroprotective bioactivities. Therefore, in the present study we wish to explore other bioactive principles of peels of *C. grandis*.

## 2. Results and Discussion

The dried peels of *C. grandis* were extracted with EtOH and the resulting extract was then filtered and concentrated. The EtOH extracts were suspended in distilled water and successively partitioned with EtOAc to afford EtOAc layer and water layer, respectively. A sequential combination of conventional chromatographic techniques was utilized to isolate the constituents described below. Totally 40 known compounds were isolated and identified, including seventeen coumarins (7-geranyloxycoumarin (**1**) [35], osthenol (**2**) [36], isomeranzin (**3**) [37], marmin (**4**) [37], epoxyaurapten (**5**) [38], meranzin hydrate (**6**) [39], hopeyhopin (**7**) [40], 7-hydroxy-8- (2′-hydroxy-3′-methylbut-3′-enyl)coumarin (**8**) [37], isoimperatorin (**9**) [41], bergamottin (**10**) [42], bergaptol (**11**) [43], 17,18-dihydroxybergamottin (**12**) [44], epoxybergamottin (**13**) [45], auraptenol (**14**) [39], columbianetin (**15**) [36], yuehgesin-C (**16**) [46], bergomottin (**17**) [47]); eight flavonoids (naringin (**18**) [48], rhoifolin (**19**) [49], naringenin 7-rutinoside (**20**) [50], melitidin (**21**) [51], vitexicarpin (**22**) [52], chrysosplin (**23**) [53], 5-hydroxy-3,6,7,3′,4′-pentamethoxyflavone (**24**) [54], rubranonoside (**25**) [55]); two triterpenoids, friedelin (**26**) [56] and limonin (**27**) [48]; four benzenoids (eleutheroside B (**28**) [57], 4-hydroxy-benzaldehyde (**29**) [58], phlorin (**30**) [59], methyl 4-hydroxybenzoate (**31**) [58]); two steroids (β-sitosterol (**32**) [58], and β-sitosterol-3-*O*-β-d-glucopyranoside (**33**) [58]); one lignan, syringaresinol (**34**) [60]; one amide, 2-hydroxybenzoic acid *N*-2-(4-hydroxyphenyl)ethylamide (**35**) [61]; and five others compounds (octadecatrienoic acid (**36**) [62], *myo*-inositol (**37**) [63], *scyllo*-inositol (**38**) [63], 1-methoxy-β-carboline (**39**) [64], and adenosine (**40**) [65]), respectively. These compounds were subjected to spectroscopic elucidation, including UV, IR, 1D- and 2D-NMR data, along with the mass spectrometric analysis and their structures were identified by comparison of their physical and spectroscopic data with values reported in the literature.

**Figure 1:**
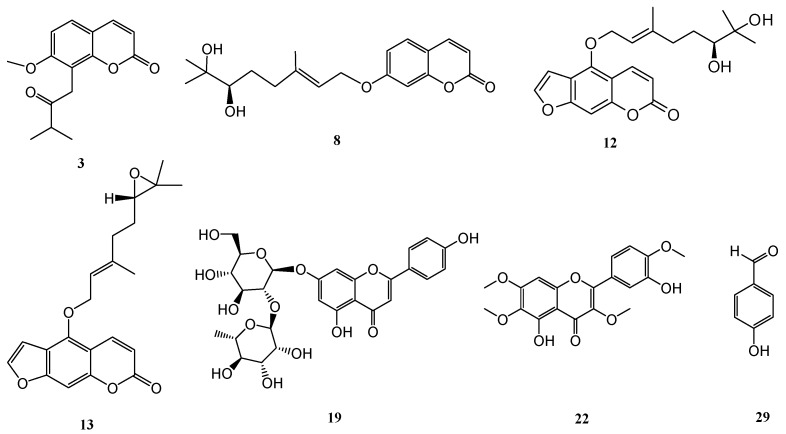
Structures of compounds **3**, **8**, **12**, **13**, **19**, **22**, and **29**.

Most of the purified compounds were examined for their inhibition of superoxide anion generation and elastase release by human neutrophils in response to *N*-formyl-l-methionyl-phenylalanine/cytochalasin B (fMLP/CB) [66,67]. Only compounds **3**, **12**, **13**, **19**, **22**, and **29** (Figure 1) displayed inhibition percentages greater than 50% at the test concentration of 10 μM and in the concentration range used these compounds displayed inhibitory effects in a dose-dependent manner. Compounds **3**, **12**, **13**, **19**, **22**, and **29** all displayed inhibition of superoxide anion generation with IC_50_ values ranging from 0.54 ± 0.24 to 7.57 ± 3.19 μM, compared to the reference compound sorafenib [66] (IC_50_ values of 1.49 ± 0.42 μM, Table 1). In addition, compounds **3**, **13**, and **29** also exhibited inhibitory effects on elastase release, with IC_50_ values ranging from 0.43 ± 0.09 to 4.33 ± 0.83 μM, compared to the reference compound sorafenib [66] (IC_50_ values of 0.93 ± 0.10 μM, Table 1).

**Figure 2:**
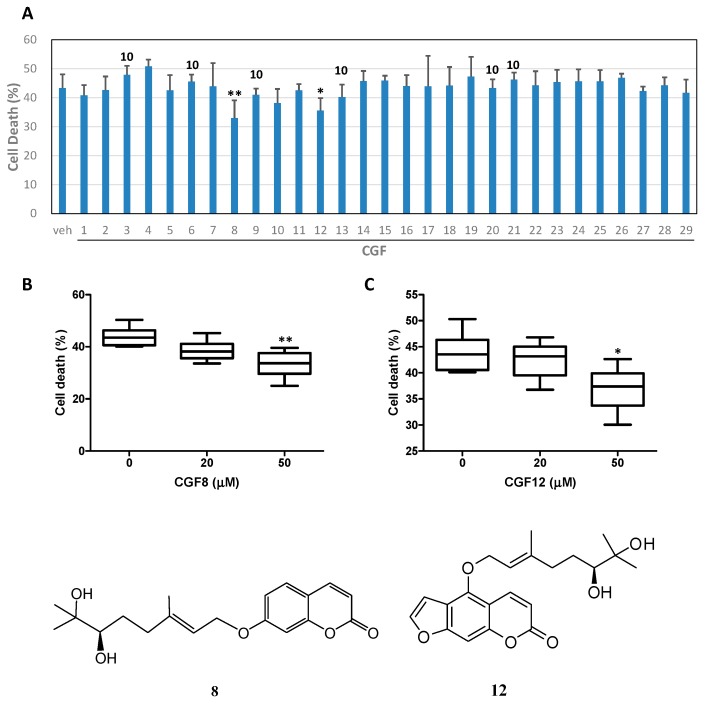
Compounds **8** and **12** protect cortical neurons against Aβ-(25–35)-mediated neurotoxicity. Cortical neurons were pretreated with 50 μM of compounds (or 10 μM for those indicated as 10 above column). (**A**), or various concentration of compounds **8** (**B**) and **12** (**C**) for 2 h and then exposed to 10 μM of Aβ-(25–35) for 40 h. Cell viability was measured by MTT reduction assay. Results are means ± S.D. from six independent experiments and expressed relative to control and plotted as box plot for showing the normal distribution. Significant differences between cells treated with Aβ-(25–35) plus vehicle (veh) and Aβ-(25–35) plus compounds are indicated by *, *p* < 0.05; **, *p* < 0.01.

In the primary screening of the neuroprotective activity of the isolated compounds, 50 μM of the compound was used. Alternatively, 10 μM of the compound was used as this compound possessed neurotoxicity at 20 μM (Figure 2A) [68]. The results showed that 7-hydroxy-8-(2′-hydroxy-3′-methylbut-3′-enyl)coumarin (**8**) and 17,18-dihydroxybergamottin (**12**) (Figure 1) protected neurons against Aβ-mediated neurotoxicity. The concentration dependency of neurons protection was further examined for compounds **8** and **12** and the experimental results displayed a dose-dependent manner as shown in Figure 2B,C.

The present experimental data suggest that the extracts and purified compounds of the peel of *C. grandis* have the potential to be developed as novel anti-inflammatory lead drugs or health foods. In addition, some purified constituents also exhibited the protection of neurons against Aβ-mediated neurotoxicity. It merits further investigations of the anti-inflammatory and neuroprotective mechanism of these natural heterocyclic compounds.

## 3. Materials and Methods

### 3.1. General Information

Melting points were determined using an MP-S3 apparatus (Yanaco, Tokyo, Japan). UV spectra were recorded at room temperature on a U-0080-D spectrophotometer (Hitachi, Tokyo, Japan). IR spectra were obtained with a FT-IR Spectrum RX I spectrophotometer (PerkinElmer, Waltham, MA, USA). Optical rotations were measured using a P-2000 digital polarimeter (JASCO, Tokyo, Japan). ^1^H- and ^13^C-NMR spectra were recorded on Avance III HD 700 and Avance III 400 NMR spectrometers (Bruker, Billerica, MA, USA). Chemical shifts are shown in δ values (ppm) with tetramethylsilane as an internal standard. The ESIMS and HRESIMS were taken on a Bruker Daltonics APEX II 30e spectrometer (positive-ion mode). Column chromatography (CC) was performed on silica (70–230 mesh and 230–400 mesh, Merck, Darmstadt, Germany), Diaion HP-20 (Mitsubishi, Tokyo, Japan), and C_18_ (Sigma-Aldrich, St. Louis, MO, USA) gels, respectively, and preparative TLC (thin-layer chromatography) was conducted on Merck precoated silica gel 60 F254 plates, using UV light to visualize the spots. High-performance liquid chromatography (HPLC) was performed on an LC-20AT series pumping system (Shimadzu, Kyoto, Japan) equipped with a Shimadzu SPD-20A UV-vis detector, and a SIL-10AF auto-sampling system at ambient temperature.

### 3.2. Materials

The peels of *C. grandis* were bought from the market in Tainan, Taiwan in 2010 and identified by Prof. Chang-Sheng Kuoh, Department of Life Science, National Cheng Kung University (NCKU), Tainan, Taiwan. A voucher specimen (Wu-2010009) was stored in School of Pharmacy, NCKU.

### 3.3. Extraction and Isolation

The dried peels of *C. grandis* (1.0 kg) were pre-treated with EtOH for one day at room temperature and then extracted with 95% EtOH five times (10 L × 5 h) at room temperature. The resulting extract was then filtered and concentrated *in vacuo* to yield a crude extract. The EtOH extract (260 g) was suspended in distilled water and successively partitioned with EtOAc yielding an EtOAc layer (20 g) and water layer (240 g). The EtOAc-soluble fraction was subjected to silica gel CC using a gradient solvent system of *n*-hexane–acetone (8:1, 4:1, 1:1 and MeOH) to afford ten fractions (F1~F10) according to TLC monitoring. Recrystallization of water layer produced the solids *myo*-inositol (**37**) (10.3 mg) and *scyllo*-inositol (**38**) (8.2 mg). The mother liquid of water layer was applied to CC on Diaion HP-20 gel using a gradient of increasing MeOH in H_2_O to yield eight fractions (W1~W8).

Fractions F1 and F2 were combined and isolated by CC on silica gel with a step gradient with *n*-hexane and diisopropyl ether mixtures (11:1, 9:1, 5:1, 2:1, 1:1, 0:1) to afford three subfractions (F1-1~F1-3). F1-2 was further purified by silica gel CC and recrystallization to obtain friedelin (**26**) (10.3 mg). Fractions F3 and F4 were combined and performed silica gel CC with gradient mixtures of *n*-hexane and ethyl acetate to produce five subfractions (F3-1~F3-5). F3-2 was purified by silica gel CC by gradient elution of *n*-hexane and ethyl acetate to afford several additional fractions. Further purification by TLC using *n*-hexane-ethyl acetate (9:1) and recrystallization yielded isoimperatorin (**9**) (16.7 mg) and β-sitosterol (**32**) (265.7 mg). F3-4 was further purified by silica gel CC and recrystallization to obtain bergomottin (**17**) (2.5 mg) and octadecatrienoic acid (**36**) (10.4 mg).

Fraction F5 was subjected to silica gel CC with diisopropyl ether as eluent to afford six subfractions (F5-1~F5-6). F5-2 was purified by silica gel CC eluted by a gradient of *n*-hexane and diisopropyl ether to afford several minor fractions. Further purification by TLC using *n*-hexane and diisopropyl ether (3:1) and recrystallization yielded osthenol (**2**) (2.6 mg), isomeranzin (**3**) (3.2 mg), marmin (**4**) (1.1 mg), and 7-hydroxy-8-(2′-hydroxy-3′-methylbut-3′-enyl)coumarin (**8**) (2.5 mg). Recrystallization of minor fractions of F5-4 produced yuehgesin-C (**16**) (302.2 mg). F5-5 was subjected to silica gel CC eluted with a gradient of *n*-hexane and ethyl acetate to afford three subfractions (F5-5-1~F5-5-3). F5-5-2 was further isolated by silica gel CC, eluted with *n*-hexane-isopropyl ether (2:1) and subsequent preparative TLC using *n*-hexane-acetone (9:1) or recrystallization of the minor fractions to afford epoxybergamottin (**13**) (1.0 mg), columbianetin (**15**) (6.6 mg), and 5-hydroxy-3,6,7,3′,4′-pentamethoxyflavone (**24**) (1.3 mg), respectively.

Fraction F7 was purified by silica gel CC eluted by a mixture of chloroform and acetone (9:1) to yield six subfractions (F7-1~F7-6). Recrystallization of subfractions F7-1 to F7-4 produced limonin (**27**) (98.3 mg), bergaptol (**11**) (7.0 mg), 17,18-dihydroxybergamottin (**12**) (22.2 mg), auraptenol (**14**) (13.5 mg), respectively. F7-5 was isolated on silica gel CC by gradient elution with *n*-hexane and acetone to afford several minor fractions. Further purification by TLC using chloroform and acetone (5:1) and recrystallization yielded vitexicarpin (**22**) (6.2 mg) and chrysosplin (**23**) (3.2 mg). Recrystallization of minor fractions of F7-6 produced *N*-[8′-(4′-hydroxyphenylethyl)]-2-hydroxy-benzoylamide (**35**) (4.0 mg).

Fractions F8 and F9 were combined and isolated by silica gel CC using a mixture of diisopropyl ether and ethyl acetate (1:1) to result in three subfractions (F8-1~F8-3). F8-2 was purified by silica gel CC with gradient elution using diisopropyl ether and ethyl acetate to afford several minor fractions. Further purification by TLC using diisopropyl ether and acetone (3:1) and recrystallization yielded meranzin hydrate (**6**) (2.8 mg), methyl 4-hydroxybenzoate (**31**) (2.1 mg), and syringaresinol (**34**) (1.9 mg).

Fractions W2 and W3 were combined and purified using C_18_ gel CC eluted with a gradient of H_2_O and MeOH (1:0, 9:1, 5:1, 2:1, 1:1, 0:1) to afford three subfractions (W2-1~W2-3). W2-3 was isolated by HPLC with an Agilent semi-preparative RP-18 column (21.2 mm × 250 mm) eluted with H_2_O and MeOH (60:40) to afford melitidin (**21**) (42.9 mg) and rubranonoside (**25**) (3.8 mg).

Fraction W4 was subjected to C_18_ gel CC eluted with a gradient of H_2_O and MeOH to yield five subfractions (W4-1~W4-5). W4-2 was subjected to HPLC with an Agilent analytical RP-18 column (4.6 mm × 250 mm) eluted with H_2_O and MeOH (55:45) to furnish rhoifolin (**19**) (3.4 mg) and naringenin 7-rutinoside (**20**) (3.0 mg). W4-3 was isolated by C_18_ gel CC with gradient mixtures of H_2_O and MeOH to afford several minor fractions. W4-3-2 was purified by HPLC on an Agilent analytical RP-18 column (4.6 mm × 250 mm) eluted with H_2_O and MeOH (70:30) to yield hopeyhopin (**7**) (3.8 mg) and eleutheroside B (**28**) (1.8 mg). Recrystallization of minor fractions of W4-4 resulted in adenosine (**40**) (13.9 mg).

Fractions W5 and W6 were combined and purified using C_18_ gel CC eluted with mixtures of H_2_O and MeOH to afford four subfractions (W5-1~W5-4). Recrystallization of subfraction W5-1 produced naringin (**18**) (440.0 mg). W5-2 was isolated by C_18_ gel CC with gradient mixtures of H_2_O and MeOH to afford phlorin (**30**) (26.3 mg). W5-3 was purified by HPLC on an Agilent analytical RP-18 column (4.6 mm × 250 mm) eluted with H_2_O and MeOH (65:35) to yield epoxyaurapten (**5**) (1.0 mg) and 1-methoxy-β-carboline (**39**) (1.0 mg).

Fraction W7 was subjected to C_18_ gel CC with gradient elution using H_2_O and MeOH to provide three subfractions (W7-1~W7-3). Recrystallization of subfractions W7-1 and W7-2 produced β-sitosterol-3-*O*-β-d-glucopyranoside (**33**) (11.6 mg) and 7-geranyloxycoumarin (**1**) (4.0 mg), respectively. Further purification of W7-3 by preparative TLC using chloroform and acetone (5:1) and recrystallization yielded 4-hydroxybenzaldehyde (**29**) (1.9 mg).

### 3.4. Bioactivity Examination

#### 3.4.1. Preparation of Human Neutrophils

A study involving human neutrophils was approved by the Institutional Review Board at Chang Gung Memorial Hospital, Taoyuan, Taiwan, and was conducted according to the Declaration of Helsinki (2013). The written informed consent was obtained from each healthy donor before blood was drawn. Blood was drawn from healthy human donors (20–30 years old) by venipuncture into heparin-coated vacutainer tubes, using a protocol approved by the Institutional Review Board at Chang Gung Memorial Hospital. Blood samples were mixed gently with an equal volume of 3% dextran solution. Neutrophils were isolated with a standard method of dextran sedimentation prior to centrifugation in a Ficoll Hypaque gradient and hypotonic lysis of erythrocytes. The leukocyte-rich plasma was collected after sedimentation of the red cells for 30 min at room temperature, and was transferred to 20 mL Ficoll solution (1.077 g/mL) and spun down at 400 g for 40 min at 20 °C. The granulocyte/erythrocyte pellets were resuspended in ice-cold 0.2% NaCl to lyse erythrocytes. After 30 s, the same volume of 1.6% NaCl solution was added to reconstitute the isotonic condition. Purified neutrophils were pelleted and then resuspended in a calcium (Ca^2+^)-free Hank’s balanced salt solution (HBSS) buffer at pH 7.4, and were maintained at 4 °C before use.

#### 3.4.2. Inhibition of Superoxide Anion Generation

The assay of the generation of superoxide anion was based on the SOD-inhibitable reduction of ferricytochrome c [65,66]. In brief, after supplementation with 0.5 mg/mL ferricytochrome c and 1 mM Ca^2+^, neutrophils (6 × 10^5^ cells/mL) were equilibrated at 37 °C for 2 min and incubated with drugs or an equal volume of vehicle (0.1% DMSO, negative control) for 5 min. Cells were activated with 100 nM fMLP during the preincubation of 1 µg/mL cytochalasin B (fMLP/CB) for 3 min. Changes in the absorbance with a reduction in ferricytochrome c at 550 nm were continuously monitored in a double-beam, six-cell positioner spectrophotometer with constant stirring (Hitachi U-3010). Calculations were based on differences in the reactions with and without SOD (100 U/mL) divided by the extinction coefficient for the reduction of ferricytochrome c (ε = 21.1/mM/10 mm at the concentration of 1 mM in cuvette with 1-cm optical path length).

#### 3.4.3. Inhibition of Elastase Release

Degranulation of azurophilic granules was determined by elastase release as described previously [65,66]. Experiments were performed using MeO-Suc-Ala-Ala-Pro-Val-*p*-nitroanilide as the elastase substrate. Briefly, after supplementation with MeO-Suc-Ala-Ala-Pro-Val-*p*-nitroanilide (100 µM), neutrophils (6 × 10^5^/mL) were equilibrated at 37 °C for 2 min and incubated with test compounds or an equal volume of vehicle (0.1% DMSO, negative control) for 5 min. Cells were activated by 100 nM fMLP and 0.5 µg/mL cytochalasin B, and changes in absorbance at 405 nm were continuously monitored to assay elastase release. The results were expressed as the percent of elastase release in the fMLP/CB-activated, drug-free control system.

#### 3.4.4. Statistical Analysis

Normal distribution with Shapiro-Wilk was performed. The results are expressed as the mean ± SD. and were analyzed by analysis of variance (ANOVA) with post-hoc Bonferroni multiple comparisons tests. Calculations of 50% inhibitory concentrations (IC_50_) were computer-assisted (PHARM/PCS v.4.2). Statistical comparisons were made between groups using the Student’s *t* test. Values of *p* less than 0.05 were considered to be statistically significant.

#### 3.4.5. Neuroprotective Activity

Primary cultures of neonatal cortical neurons were prepared from the cerebral cortex of Harlan Sprague-Dawley rat pups at postnatal day 1 [67]. Briefly, each pup was decapitated and the cortex was digested in 0.5 mg/mL papain at 37 °C for 15 min. The tissue was dissociated in Hypernate A medium (containing B27 supplement) by aspirating trituration. Cells were plated (5 × 10^4^ cells/cm^2^) onto poly-d-lysine-coated dishes and maintained in neurobasal medium containing B27 supplement, 10 units/mL penicillin, 10 mg/mL streptomycin, and 0.5 mg/mL glutamine (5% CO_2_/9% O_2_) for 3 days. Cells were then exposed to cytosine-β-D-arabinofuranoside (5 mM) for 1 day to inhibit proliferation of non-neuronal cells. The cells were used for the experiment on the fifth day. The reduction of MTT was used to evaluate the cell viability. Cells were incubated with minimum essential medium containing 0.5 mg/mL MTT for 1 h. The medium was aspirated, and the formazan particle was dissolved with lysis buffer (10% sodium dodecyl sulfate, 3.3 mM HCl, 50% dimethylformamide). A 600 nm absorbance was measured by using enzyme-linked immunosorbent assay reader. The results are expressed as the mean ± SD and were analyzed by analysis of variance (ANOVA) with post-hoc Bonferroni multiple comparisons tests.

## Figures and Tables

**Table 1:** Inhibitory effects of isolated compounds on superoxide anion generation and elastase release by human neutrophils in response to fMLP/CB.

Compound	Superoxide Anion Generation	Elastase Release
	IC_50_ (μM) ^a^	IC_50_ (μM) ^a^
**3**	3.89 ± 0.45 ***	4.33 ± 0.83 ***
**12**	6.02 ± 2.46 ***	>10
**13**	7.57 ± 3.19 ***	3.58 ± 1.90 ***
**19**	3.79 ± 0.42 ***	>10
**22**	5.95 ± 1.56 ***	>10
**29**	0.54 ± 0.24 ***	0.43 ± 0.09 ***
Sorafenib ^b^	1.49 ± 0.42	0.93 ± 0.10

^a^ Concentration necessary for 50% inhibition (IC_50_). Results are presented as means ± SD. (*n* = 3~5). *** *p* < 0.001 compared with the control value. ^b^ Sorafenib, a tyrosine kinase inhibitor, was used as a positive control.
